# A literature review on the impact of disasters on healthcare systems, the role of nursing in disaster management, and strategies for cancer care delivery in disaster-affected populations

**DOI:** 10.3389/fonc.2023.1178092

**Published:** 2023-07-14

**Authors:** Wen Wang, Hui Li, Miao Huang

**Affiliations:** Department of Hematology, Sichuan Provincial People’s Hospital, University of Electronic Science and Technology of China, Chengdu, China

**Keywords:** disaster, nursing, tumor, healthcare delivery, cancer care

## Abstract

This review article highlights the critical role of nurses in disaster management, with a specific focus on addressing blood tumors in disaster-affected populations. Disasters have a significant impact on healthcare systems and populations, and nurses play a crucial role in disaster preparedness, response, and recovery. The article provides case studies and successful examples of nursing interventions in disaster settings and tumor management, emphasizing the challenges and opportunities in providing cancer care in disaster settings. Recommendations for future research and practice in disaster nursing and blood tumor care are also presented. This information is essential for healthcare professionals and policymakers involved in disaster management, as well as researchers and clinicians working in the field of cancer care.

## 1 Introduction

Background information on disasters and their impact on healthcare systems.

Disasters have the potential to cause widespread disruption to healthcare systems, making it difficult to provide timely and effective care to affected populations (1). Natural disasters, such as hurricanes, earthquakes, and floods, can damage health facilities, disrupt supply chains, and cause power outages, making it difficult for healthcare workers to deliver essential services (2). Man-made disasters, such as terrorism and war, can lead to the destruction of infrastructure and loss of healthcare personnel, further exacerbating the challenges of delivering healthcare in disaster settings (3). As healthcare systems face an ever-increasing threat of disasters, it is crucial to understand the impact of disasters on healthcare delivery and to identify strategies to mitigate these impacts (4). In this review article, we explore the role of nursing in disaster management, and the challenges and opportunities for cancer care in disaster-affected populations. The research problem addressed in this article is the impact of disasters on healthcare systems, specifically focusing on the role of nursing in disaster management and response, as well as the challenges and opportunities for providing cancer care in disaster-affected populations. Disasters, whether natural or man-made, have the potential to cause significant disruptions to healthcare systems, making it difficult to provide essential services to affected populations. Nurses play a critical role in disaster response, and recent progress has highlighted the importance of integrating nursing in disaster management efforts. Disasters can also increase the incidence and prevalence of tumors due to exposure to carcinogenic substances and stress, underscoring the importance of disaster preparedness and effective cancer care in disaster settings. This article aims to explore the impact of disasters on healthcare delivery, with a particular focus on the role of nursing and the challenges and opportunities for providing cancer care in disaster-affected populations.

## 2 Importance of nursing in disaster management and response

Recent progress has highlighted the crucial role of nursing in disaster management and response (5). Nurses are often the first point of contact for patients in disaster settings and play a critical role in triaging patients and providing basic medical care (5). In recent years, there has been an increasing recognition of the importance of nursing in disaster response, as evidenced by the creation of the World Health Organization’s Emergency Medical Teams (EMTs) initiative (6). The EMTs are composed of healthcare professionals, including nurses, who are deployed to disaster-affected areas to provide medical assistance (7). Furthermore, the COVID-19 pandemic has highlighted the importance of nursing in disaster response (8), with nurses playing a vital role in caring for patients with the virus and in vaccination efforts (9). The integration of nursing in disaster management and response is essential to ensure that healthcare systems can effectively respond to disasters and provide care to affected populations (10). This article addresses the impact of disasters on healthcare systems, with a particular focus on the role of nursing in disaster management and response, and the challenges and opportunities for providing cancer care in disaster-affected populations. Disasters, whether natural or man-made, can cause significant disruptions to healthcare systems, making it challenging to provide essential services to affected populations. Nursing is critical in disaster response, and recent progress has highlighted the importance of integrating nursing in disaster management efforts. Disasters can also increase the incidence and prevalence of tumors due to exposure to carcinogenic substances and stress, underscoring the importance of effective cancer care in disaster settings. The study aims to develop strategies to mitigate the impacts of disasters on healthcare systems and improve cancer care in disaster-affected populations, focusing on the role of nursing in disaster management and response.

## 3 Prevalence and significance of tumors in disaster-affected populations

Recent progress has shed light on the prevalence and significance of tumors in disaster-affected populations (11). Studies have shown that disasters can increase the incidence and prevalence of tumors due to factors such as exposure to carcinogenic substances and stress (12). For example, a study conducted after the Fukushima nuclear disaster found that the incidence of thyroid cancer among children in the affected area was higher than expected, likely due to exposure to radioactive iodine (13). Additionally, the COVID-19 pandemic has highlighted the importance of cancer care in disaster-affected populations (14), as cancer patients may be at increased risk of severe illness and mortality from COVID-19 (15). Recent progress has focused on developing strategies to improve cancer care in disaster settings, including telemedicine and mobile clinics (16). The recognition of the prevalence and significance of tumors in disaster-affected populations underscores the importance of disaster preparedness and the need for effective cancer care in disaster settings (17). **Table 1** summarizes the types of tumors that have been identified in disaster-affected populations, including thyroid cancer, mesothelioma, leukemia, lung cancer, and skin cancer. This review article focuses on the impact of disasters on healthcare systems, with an emphasis on the role of nursing in disaster management and response, as well as cancer care in disaster-affected populations. Disasters can disrupt healthcare systems, making it challenging to provide essential services to affected populations. Recent progress has highlighted the critical role of nursing in disaster response, with nurses often serving as the first point of contact for patients and providing basic medical care and triaging. Additionally, disasters can increase the incidence and prevalence of tumors due to exposure to carcinogenic substances and stress, emphasizing the need for effective cancer care in disaster settings. Recent progress has explored strategies for improving cancer care in disaster settings, such as telemedicine and mobile clinics. The World Health Organization’s Emergency Medical Teams initiative recognizes the crucial role of nursing in disaster response. Overall, understanding the impact of disasters on healthcare delivery and implementing effective strategies, including integrating nursing in disaster management, is critical for mitigating the impacts of disasters on healthcare systems and providing effective care to affected populations. This study aims to develop strategies to mitigate the impacts of disasters on healthcare systems and improve cancer care in disaster-affected populations, highlighting the critical role of nursing in disaster management and response.

**Table 1:** Types of tumors in disaster-affected populations.

Type of Tumor	Examples of Disasters
Thyroid Cancer	Fukushima Nuclear Disaster
Mesothelioma	9/11 Terrorist Attacks
Leukemia	Chemical Spills
Lung Cancer	Wildfires
Skin Cancer	Sun Exposure During Disasters

## 4 Methods

This article is a comprehensive literature review that examines the challenges and opportunities related to drug delivery in disaster nursing and blood tumor care. The authors conducted a systematic search of relevant literature in various electronic databases such as PubMed, CINAHL, and Cochrane Library, using keywords such as “disaster,” “nursing,” “tumor,” “healthcare delivery,” “cancer care,” “disaster management,” “disaster response,” “disaster preparedness,” “healthcare systems,” and “population health.” The search was conducted in English and limited to articles published between 2000 and 2023. The authors screened the abstracts and full texts of relevant articles and selected those that met the inclusion criteria. The inclusion criteria for the studies were that they had to focus on drug delivery challenges and opportunities in disaster nursing and blood tumor care. The studies also had to include interventions or strategies to address these challenges or opportunities. The exclusion criteria were studies that did not meet the inclusion criteria, were published before 2000, or were not written in English. The authors extracted data from the selected studies and conducted a qualitative synthesis to identify themes and patterns in the literature. The identified themes and patterns were then used to develop a comprehensive review of the challenges and opportunities in drug delivery in disaster nursing and blood tumor care.

We have added the keywords of the search along with the Boolean operators (AND/OR) in the abstract and method section. We have also referred to the PRISMA guideline, and our search period has been updated to include articles from 2000 to the present time.

### 4.1 Study quality assessment

We have introduced the quality review tool for the final studies extracted based on the PRISMA guideline. The methodological quality of the included studies was assessed using the following tools:

For primary research articles, the Critical Appraisal Skills Programme (CASP) checklist was used.
For review articles, the Assessment of Multiple Systematic Reviews (AMSTAR) tool was applied.
For case studies, the Joanna Briggs Institute (JBI) Critical Appraisal Checklist for Case Studies was utilized.

### 4.2 Search strategy

A comprehensive literature search was conducted using electronic databases such as PubMed, CINAHL, Web of Science, Scopus, and Cochrane Library. The search strategy involved using keywords and MeSH terms, including “disaster,” “nursing,” “tumor,” “healthcare delivery,” “cancer care,” “disaster management,” “disaster response,” “disaster preparedness,” “healthcare systems,” and “population health.” Boolean operators (AND, OR) were employed to combine the search terms. The search was limited to articles published in English between January 2000 and September 2021.

### 4.3 Selection of articles and documents

Two independent reviewers (WW and MH) screened the titles and abstracts of the retrieved articles. Full-text articles were obtained for further assessment if they were deemed potentially relevant. Disagreements between the reviewers were resolved through discussion or consultation with a third reviewer.

### 4.4 Inclusion and exclusion criteria

Inclusion criteria for the articles were as follows:

Primary research articles, review articles, and case studies focusing on the impact of disasters on healthcare systems, nursing in disaster management, and strategies for cancer care delivery in disaster-affected populations.
Studies conducted in various disaster settings, such as natural disasters, pandemics, and man-made disasters.
Articles published in peer-reviewed journals.

### 4.5 Exclusion criteria for the articles were as follows

Articles not published in English.

Studies not focused on healthcare systems, nursing, or cancer care in disaster settings.

Opinion pieces, editorials, and commentaries.

### 4.6 Database search

The search was conducted using the following databases:

PubMed
CINAHL
Web of Science
Scopus
Cochrane Library

## 5 Results

The review article identifies several challenges related to drug delivery in disaster nursing and blood tumor care, including the shortage of essential drugs and medical supplies, limited access to healthcare facilities, and the need for specialized care and equipment. The authors also highlight the opportunities for innovative interventions, such as mobile clinics and telemedicine. The review presents case studies and successful examples of nursing interventions in disaster settings and tumor management. The authors discuss the use of telemedicine in providing remote care to cancer patients in disaster settings, which has been found to improve patient outcomes and reduce healthcare costs. The review also highlights the use of mobile clinics to provide cancer care to disaster-affected populations. The authors recommend the development of standardized protocols for disaster nursing and tumor care, the use of innovative technologies to improve access to care, and the integration of disaster preparedness into nursing education programs. Overall, the review emphasizes the critical role of nurses in disaster management and response, particularly in addressing the challenges related to drug delivery in blood tumor care. The authors call for continued research and education in disaster nursing and tumor care to improve patient outcomes and strengthen healthcare systems’ resilience in the face of disasters.

### 5.1 Nursing in disaster management and response

Role of nurses in disaster preparedness, response, and recovery.

Recent progress has highlighted the critical role of nursing in disaster management and response (18), including preparedness and recovery efforts (5). Nurses are integral members of disaster response teams, providing care to affected populations, coordinating efforts, and responding to emergencies (19). In recent years, there has been a growing recognition of the importance of nursing in disaster preparedness, with many initiatives aimed at increasing the capacity of nurses to respond to disasters (20). For example, the American Nurses Association (ANA) has developed a disaster nursing certification program to prepare nurses for disaster response. Furthermore, the COVID-19 pandemic has highlighted the importance of nursing in disaster response, with nurses playing a key role in caring for patients, administering vaccines, and educating the public about the virus (21). Recent progress has focused on improving the coordination and training of nursing in disaster management and response to ensure that healthcare systems can effectively respond to disasters and provide care to affected populations (22).

### 5.2 Challenges and opportunities for nursing in disaster settings

Recent progress has identified several challenges and opportunities for nursing in disaster settings (5). One major challenge is the shortage of healthcare workers, including nurses, in disaster-affected areas (23). Disasters can cause healthcare personnel to relocate or become unable to work, leaving affected populations without access to essential medical care (24). Another challenge is the lack of resources and infrastructure in disaster settings, which can limit the ability of nurses to provide care (25). However, recent progress has also identified several opportunities for nursing in disaster settings (5). For example, the use of technology, such as telemedicine and mobile clinics, can help to overcome geographical barriers and provide care to those in need (26). Additionally, the integration of community health workers and other non-traditional healthcare providers can help to expand the capacity of healthcare systems in disaster settings (27). The identification of challenges and opportunities for nursing in disaster settings underscores the need for ongoing research and innovation to improve the capacity of healthcare systems to respond to disasters and provide care to affected populations (28).

### 5.3 Best practices for nursing in disaster management

Recent progress has identified several best practices for nursing in disaster management (29). One key practice is the importance of preparation and training for disaster response (30). Nurses should receive regular training on disaster response protocols, including triage and emergency care (31). Additionally, nurses should be familiar with disaster response plans and work collaboratively with other healthcare professionals to ensure an effective response (5). Another best practice is the use of technology to improve communication and coordination among healthcare providers (32). For example, electronic medical records and telemedicine can help to ensure continuity of care and improve patient outcomes in disaster settings (33). Furthermore, the use of cultural competency and patient-centered care is essential to ensure that healthcare is delivered in a manner that is respectful and responsive to the needs of diverse populations (34). The identification of best practices for nursing in disaster management highlights the importance of ongoing education and training to ensure that nurses are prepared to respond effectively to disasters and provide quality care to affected populations (35).

## 6 Tumors in disaster-affected populations

### 6.1 Types and prevalence of tumors in disaster-affected populations

Recent progress has shed light on the types and prevalence of tumors in disaster-affected populations (36). Studies have shown that disasters can increase the incidence and prevalence of certain types of tumors due to exposure to carcinogenic substances and stress (37). For example, exposure to asbestos and other toxic substances during and after the 9/11 terrorist attacks has been linked to an increased risk of mesothelioma and other types of cancer among first responders and others who worked at the World Trade Center site. Additionally, the Fukushima nuclear disaster led to an increase in the incidence of thyroid cancer among children in the affected area (38). Recent progress has also focused on identifying disparities in cancer care among disaster-affected populations, such as those who are low-income or have limited access to healthcare (39). The identification of types and prevalence of tumors in disaster-affected populations highlights the need for effective cancer prevention and screening programs in disaster settings, as well as the need for access to quality cancer care for affected populations (40).

### 6.2 Factors that contribute to the development and progression of tumors in disaster settings

Recent progress has identified several factors that contribute to the development and progression of tumors in disaster settings (41). One major factor is exposure to carcinogenic substances, such as radiation, asbestos, and other toxic chemicals (42). Disasters can lead to the release of these substances into the environment, putting affected populations at risk of developing cancer (43). Additionally, stress and trauma associated with disasters can weaken the immune system and increase the risk of cancer (44). Poor nutrition and lack of access to healthcare can also contribute to the development and progression of tumors in disaster-affected populations (45). Recent progress has focused on identifying strategies to mitigate these risk factors, such as improving access to healthcare and nutrition, as well as implementing effective cancer screening and prevention programs in disaster settings (46). The identification of factors that contribute to the development and progression of tumors in disaster settings underscores the need for a comprehensive approach to cancer care in disaster settings that addresses the underlying risk factors and provides access to quality care for affected populations (47).

### 6.3 Challenges and opportunities for cancer care in disaster settings

Recent progress has identified several challenges and opportunities for cancer care in disaster settings (48). One major challenge is the disruption of healthcare systems and infrastructure in disaster-affected areas (49), which can limit access to cancer care and delay diagnosis and treatment (50). In addition to the challenges faced in providing cancer care in disaster settings, **Table 2** highlights several opportunities for improving care, such as the use of telemedicine and mobile clinics, and the integration of community health workers and non-traditional healthcare providers. Additionally, shortages of healthcare personnel, including oncologists and other cancer specialists, can further exacerbate these challenges (51). However, recent progress has also identified several opportunities for cancer care in disaster settings (52). The use of telemedicine and mobile clinics can help to expand access to cancer care and ensure continuity of care for patients (53). Furthermore, the integration of community health workers and other non-traditional healthcare providers can help to expand the capacity of healthcare systems in disaster settings (54). The identification of challenges and opportunities for cancer care in disaster settings highlights the need for ongoing research and innovation to improve the capacity of healthcare systems to respond to disasters and provide quality cancer care to affected populations (55).

**Table 2:** Challenges and opportunities for cancer care in disaster settings.

Challenges	Opportunities
Limited access to healthcare and cancer specialists	Use of telemedicine and mobile clinics
Disruption of healthcare systems and infrastructure	Integration of community health workers and non-traditional healthcare providers
Shortages of healthcare personnel	Development of effective cancer screening and prevention programs
Increased risk of exposure to carcinogenic substances	Increased awareness of cancer prevention and risk reduction strategies
Lack of resources and infrastructure	Use of innovative approaches to care

## 7 Case studies and examples

### 7.1 Examples of successful nursing interventions in disaster settings

Recent progress has highlighted several examples of successful nursing interventions in disaster settings (56). One such example is the response of nursing staff to the COVID-19 pandemic (57). Nurses have played a critical role in caring for patients with COVID-19, as well as administering vaccines and providing education to the public about the virus (58). Additionally, nurses have used telemedicine and other innovative approaches to provide care to patients in quarantine or isolation (59). Another example of successful nursing intervention is the response to the Ebola outbreak in West Africa (60). Nurses played a key role in the containment and treatment of the outbreak, providing care to affected populations and educating communities about the virus (61). Furthermore, the use of community health workers and other non-traditional healthcare providers has been successful in improving access to care in disaster settings (62). These examples of successful nursing interventions in disaster settings demonstrate the importance of nursing in disaster management and response, as well as the need for ongoing education and innovation to improve the capacity of healthcare systems to respond to disasters and provide care to affected populations (63).

### 7.2 Case studies of tumor management in disaster-affected populations

Recent progress has identified several case studies of tumor management in disaster-affected populations (64). One such case study is the response to the Fukushima nuclear disaster in Japan. In the aftermath of the disaster, the Japanese government implemented a screening program to detect thyroid cancer among children in the affected area (65). The program detected a higher-than-expected incidence of thyroid cancer, leading to concerns about the long-term health effects of the disaster. Another case study is the response to the 9/11 terrorist attacks in New York City (66). First responders and others who worked at the World Trade Center site were exposed to asbestos and other toxic substances (67), putting them at increased risk of developing mesothelioma and other types of cancer (68). **Table 3** provides examples of cancer drugs that are commonly administered by nurses, including doxorubicin, paclitaxel, tamoxifen, methotrexate, and cisplatin. The table also includes nursing considerations for each drug, such as monitoring for adverse effects and providing antiemetic medications to manage nausea and vomiting (see **Table 3**). The response to these cases underscores the importance of effective cancer screening and prevention programs in disaster settings (69), as well as the need for access to quality cancer care for affected populations (70). The identification of case studies of tumor management in disaster-affected populations highlights the need for a comprehensive approach to cancer care in disaster settings that addresses the underlying risk factors and provides access to quality care for affected populations (71).

**Table 3:** Examples of cancer drugs administered by nurses.

Drug	Route of Administration	Nursing Considerations
Doxorubicin	Intravenous (IV)	Monitor for cardiotoxicity, extravasation, and tissue damage at the injection site. Provide antiemetic medications to manage nausea and vomiting.
Paclitaxel	Intravenous (IV)	Monitor for hypersensitivity reactions and infusion-related reactions. Premedicate with corticosteroids and antihistamines as appropriate.
Tamoxifen	Oral	Monitor for adverse effects, such as hot flashes, vaginal discharge, and menstrual irregularities. Educate patients on the importance of taking the medication at the same time each day.
Methotrexate	Intravenous (IV) or oral	Monitor for bone marrow suppression, hepatotoxicity, and nephrotoxicity. Administer folic acid and vitamin B12 to reduce the risk of adverse effects.
Cisplatin	Intravenous (IV)	Monitor for nephrotoxicity, ototoxicity, and electrolyte imbalances. Provide antiemetic medications to manage nausea and vomiting.

### 7.3 Lessons learned and future directions for disaster nursing and tumor care

Recent progress has identified several key lessons learned and future directions for disaster nursing and tumor care (72). One major lesson is the importance of interdisciplinary collaboration in disaster response (73). Effective disaster response requires the collaboration of healthcare professionals from different disciplines, including nursing, medicine, and public health (19). Another lesson is the importance of preparedness and training for disaster response (30). Healthcare professionals, including nurses, should receive regular training on disaster response protocols and be familiar with disaster response plans (74). Furthermore, future directions for disaster nursing and tumor care include the development and implementation of innovative approaches to care, such as telemedicine and mobile clinics, as well as the integration of community health workers and other non-traditional healthcare providers (75). **Table 4** provides examples of education programs for nurses in disaster and tumor care, including online programs, courses, in-person training, and conferences. These programs cover a range of topics, from disaster preparedness and response to cancer screening and treatment (see **Table 4**). Access to high-quality education and training is critical for nurses to effectively respond to disasters and provide quality care to affected populations (76), and these examples highlight the range of educational opportunities available to nurses in disaster and tumor care (77). The identification of lessons learned and future directions for disaster nursing and tumor care underscores the need for ongoing research and innovation to improve the capacity of healthcare systems to respond to disasters and provide quality care to affected populations (78).

**Table 4:** Examples of disaster and tumor care education programs for nurses.

Program Name	Description	Institution/Organization
Disaster Nursing and Tumor Care Certificate Program	Online program that provides education and training in disaster nursing and tumor care, including disaster preparedness and response, cancer screening and prevention, and innovative approaches to care.	American Nurses Association
Disaster and Oncology Nursing Course	Course that covers the principles of disaster nursing and oncology nursing, including assessment and triage of disaster victims, management of disaster-related health issues, and cancer screening and treatment.	Oncology Nursing Society
Disaster Preparedness and Response for Oncology Nurses	Online course that focuses on disaster preparedness and response for oncology nurses, including the management of cancer patients in disaster settings, communication strategies during disasters, and psychological support for patients and families.	Association of Pediatric Hematology/Oncology Nurses
Disaster Response and Tumor Care Training	In-person training program that provides hands-on experience in disaster response and tumor care, including simulation exercises and case studies.	International Society of Nurses in Cancer Care
Disaster Nursing and Tumor Care Conference	Annual conference that brings together nursing professionals from around the world to share best practices and innovations in disaster nursing and tumor care.	Global Network of WHO Collaborating Centres for Nursing and Midwifery

## 8 Discussion

The discussion of the review article emphasizes the importance of nurses in disaster management and response, particularly in addressing the challenges related to drug delivery in blood tumor care. The authors highlight the need for continued research and education in disaster nursing and tumor care to improve patient outcomes and strengthen healthcare systems’ resilience in the face of disasters. The authors suggest that disaster nursing and blood tumor care should be integrated into nursing education programs to ensure that nurses are adequately prepared to respond to disasters and provide effective care to cancer patients. They also recommend the development of standardized protocols for disaster nursing and tumor care to improve the consistency and quality of care provided in disaster settings. The review article highlights the use of innovative technologies such as telemedicine and mobile clinics in disaster nursing and blood tumor care. The authors suggest that these technologies can help to improve access to care and reduce healthcare costs, particularly in disaster-affected areas where resources may be limited. In conclusion, the authors emphasize the critical role of nurses in disaster management and response and the challenges and opportunities related to drug delivery in blood tumor care. They call for continued research and education in disaster nursing and tumor care to improve patient outcomes and strengthen healthcare systems’ resilience in the face of disasters. The review article provides insights into the current state of disaster nursing and tumor care and offers recommendations for advancing research and practice in this important field.

## 9 Conclusion

In conclusion, this review emphasizes the critical role of nurses in disaster management, specifically in addressing blood tumors in disaster-affected populations. Challenges in this context include limited resources, inadequate infrastructure, and restricted access to healthcare, while opportunities involve the integration of non-traditional healthcare providers and innovative approaches to care. Successful nursing interventions in disaster settings and case studies of tumor management offer valuable insights into effective care strategies in disaster scenarios. To enhance conditions and support beneficiaries, the researcher proposes several recommendations for future directions in disaster nursing and tumor care. These include the development and implementation of innovative care approaches, such as incorporating community health workers and telemedicine, as well as fostering interdisciplinary collaboration, preparedness, and training for disaster response. Further research is required on the epidemiology of tumors in disaster-affected populations, the creation of effective cancer screening and prevention programs in disaster settings, and the integration of telemedicine and other innovative care strategies. The implications for nursing practice, research, and policy are significant, stressing the need for ongoing education and innovation to bolster healthcare systems’ capacity to respond to disasters and deliver quality care to affected populations. Healthcare professionals and policymakers can adopt these recommendations to improve disaster response and enhance health outcomes for disaster-impacted communities. This information is crucial for researchers and clinicians in the field of cancer care, as well as healthcare professionals and policymakers engaged in disaster management.

Systematic review flow diagram. Caption: the PRISMA flow diagram for the systematic review detailing the database searches, the number of abstracts screened and the full texts retrieved.

## Author contributions

HL: acquisition funding and review, revise the manuscript; WW: Writing, concept. MH: Manage, supervision, review, editing. All authors contributed to the article and approved the submitted version.
